# Complete Genome Sequences of Porcine Deltacoronavirus Strains DH1/2016 and DH2/2016 Isolated in South Korea

**DOI:** 10.1128/genomeA.01706-16

**Published:** 2017-05-04

**Authors:** Hee-Chun Chung, Van Giap Nguyen, Woo-Taek Oh, Huynh Thi My Le, Hyung-Joon Moon, Jee-Hoon Lee, Hye-Kwon Kim, Seong-Jun Park, Bong Kyun Park

**Affiliations:** a Department of Veterinary Medicine Virology Lab, College of Veterinary Medicine and Research Institute for Veterinary Science, Seoul National University, Seoul, Republic of Korea; b Department of Veterinary Microbiology and Infectious Diseases, Faculty of Veterinary Medicine, Vietnam National University of Agriculture, Hanoi, Vietnam; c Research Unit, Green Cross Veterinary Products, Yongin, Republic of Korea; d Viral Infectious Disease Research Center, Korea Research Institute of Bioscience and Biotechnology, Daejeon, Republic of Korea; e Forensic Medicine Division, Daegu Institute, National Forensic Service, Chilgok, Republic of Korea

## Abstract

Two porcine deltacoronavirus (PDCoV) strains, named DH1/2016 and DH2/2016, were isolated from feces of piglets which had severe watery diarrhea symptoms. A comparison of the complete genome sequences suggested that the DH1/2016 and DH2/2016 strains are highly homologous to each other and to PDCoVs isolated in early 2014 from the United States.

## GENOME ANNOUNCEMENT

Porcine deltacoronaviruses (PDCoVs) belong to the *Deltacoronavirus* genus of the *Coronaviridae* family (1). Challenging experiments in piglets with PDCoV (2) resulted in clinical symptoms similar to those of porcine epidemic diarrhea (PED). Until now, PDCoVs were reported in many countries, including Hong Kong, China, the United States, and Thailand (3–7). In South Korea, PDCoV KNU14-04 and SL strains (SL2 and SL5) had been detected in 2014 and 2015, respectively (6, 7). In this study, further complete genome sequences of the viruses isolated in 2016 were sequenced, which aimed to provide more material for the molecular analyses of PDCoVs all over the world, including in South Korea.

From May 2015 to July 2016, for diagnosis of enteric viral diseases, 687 diarrhea clinical samples (including feces and small intestines) from 9 provinces were sent to the Department of Veterinary Medicine Virology Laboratory, Seoul National University. Total RNA was extracted by using the viral RNA minikit (Qiagen Ltd., Manchester, United Kingdom), according to the manufacturer’s instructions. The RNA was then converted into cDNA with the use of random hexamers and the commercial Moloney murine leukemia virus (M-MLV) reverse transcriptase kit (Invitrogen, USA), according to the manufacturer’s protocol. Among 687 tested samples, only 5 fecal samples from Kyunggi Province (120-sow DH farm) on 5 April 2016 were PDCoV positive, but porcine epidemic diarrhea virus (PEDV), transmissible gastroenteritis coronavirus (TGEV), group A rotavirus, and kobuvirus were not detected. Retrospective investigation revealed that the pigs of all ages at the DH farm showed clinical symptoms of severe diarrhea, with mortality rates of 60% in suckling piglets and 10% in sows.

The full-length genome was sequenced by a primer walking method, which utilized 26 overlapping primer pairs (8). The specific PCR products were purified by using the gel extraction method and further processed for TA cloning and transformation (9). Two full-length genomes of the DH farm PDCoVs (DH1/2016 and DH2/2016) were characterized. The lengths of the two complete genomes were 25,422 nucleotides (nt), and they were 100% homologous to each other. The genomic organization of the DH/2016 strains was similar to that of the previously described strain KOR/KNU14-04/2014 (GenBank accession no. KM820765) (7). The full-length genome of DH/2016 strains had very high nucleotide (99.7%) and amino acid identities (99.5%) compared to the KNU14-04 strain of PDCoV. In particular, open reading frame 1a/1b (19 amino acids [aa]) and nucleocapsid (2 aa) gene substitutions at the amino acid level were observed.

According to a phylogenetic analysis based on the full-length PDCoV genomes of 65 isolates available in GenBank, the DH/2016 strains belong to a strain identified in 2014 by the U.S. groups separated from PDCoVs reported in both China and the United States. In comparison to the complete genome sequence of the viruses isolated in China (GenBank accession numbers JQ065042 and JQ065043) and the United States (GenBank accession numbers KJ462462, KJ481931, KJ567050, KJ569769, KJ601777, KJ601778, KJ601779, and KJ601780), the DH/2016 strains were also highly similar (98.8 to 99.0% and 99.6 to 99.8%, respectively).

In conclusion, our study contributed to the collection of the complete genome sequences of PDCoVs, which might be useful for molecular analyses of the virus.

### Accession number(s).

The complete genome sequences (DH1/2016 and DH2/2016) have been deposited to GenBank under the accession numbers KY354363 and KY354364.
